# Ischial osteochondroma as an unusual source of pregnancy-related sciatic pain: a case report

**DOI:** 10.1186/s12998-022-00451-3

**Published:** 2022-10-17

**Authors:** Robert J. Trager, Sarah E. Prosak, Patrick J. Getty, Richard L. Barger, Shahrazad T. Saab, Jeffery A. Dusek

**Affiliations:** 1 Connor Whole Health, University Hospitals Cleveland Medical Center, 44106 Cleveland, OH USA; 2 Musculoskeletal Surgical Oncology, University Hospitals Cleveland Medical Center, 44106 Cleveland, OH USA; 3 Orthopaedic Surgery, Case Western Reserve University, 44106 Cleveland, OH USA; 4 Division of Musculoskeletal Radiology, University Hospitals Cleveland Medical Center, 44106 Cleveland, OH USA; 5 Department of Pathology, Case Western Reserve University School of Medicine, 44106 Cleveland, OH USA; 6 Department of Family Medicine and Community Health, School of Medicine, Case Western Reserve University, 44106 Cleveland, OH USA

**Keywords:** Osteochondroma, Bone neoplasms, Chiropractic, Sciatica, Sciatic neuropathy, Low back Pain

## Abstract

**Background:**

While most cases of sciatica result from degenerative conditions of the low back, some cases result from conditions of the hip and pelvic region. Sciatica developing in relation to pregnancy or labor also presents unique considerations.

**Case presentation:**

A 37-year-old African American woman with a history of hypertension and polycystic ovary syndrome presented to a chiropractor at a hospital-based outpatient clinic with a seven-week history of low back pain with radiation into the right lower extremity which began during labor. The chiropractor performed a brief trial of care, yet when the patient’s symptoms worsened, ordered lumbar spine radiographs, followed by lumbar magnetic resonance imaging (MRI), which were both normal. The chiropractor then ordered hip radiographs, which were suggestive of ischial osteochondroma, and referred the patient to an orthopedic oncologist. MRI findings were compatible with an osteochondroma with associated adventitial bursitis and mass effect on the sciatic nerve. The patient initially chose conservative management with bursa aspiration and therapeutic injection. Despite initial relief, there was eventual return of symptoms. The patient elected to undergo surgical removal, with a positive outcome.

**Conclusion:**

The key distinguishing features that led to a diagnosis of osteochondroma in this case included attention to the patient-reported symptoms and history, worsening of symptoms despite conservative care, and lack of explanatory findings on lumbar imaging. This case highlights the benefit of evaluating the hip and pelvis when the clinical features of sciatica cannot be ascribed to a lumbar etiology. This case also illustrates the role of a chiropractor working in an integrative health system to facilitate timely imaging and referrals to resolve a challenging diagnosis.

**Supplementary information:**

The online version contains supplementary material available at 10.1186/s12998-022-00451-3.

## Background

Sciatica, defined as pain along the course and distribution of the sciatic nerve, occurs in about 1% of pregnant women [1]. In the general population lumbar disc herniation is the most common cause of sciatica [2]. However, lumbar disc herniation is uncommon during pregnancy, affecting only one in 10,000 women [3]. Accordingly, atypical causes of sciatica, including those outside of the lumbar spine, should be considered in women developing these symptoms during pregnancy or postpartum.

About one to three percent of women develop an *obstetric palsy*, a lumbosacral or lower extremity nerve injury related to labor [4, 5]. There are several potential explanations for this condition: direct pressure from the fetal head against the lumbosacral plexus, sciatic nerve stretch related to the lithotomy position (supine with hips and knees flexed), sacroiliac joint disorders, sacral stress fracture, and deep venous thrombosis [5–8]. Trauma to the spinal cord due to epidural analgesia is also a rare consideration in sciatica developing after labor [9].

There are several challenges with identifying the atypical sources of sciatica that may develop during pregnancy or labor. Clinical features can resemble those of lumbar disc herniation [7], and may be misdiagnosed [5]. Imaging is often delayed until after delivery, especially in the case of ionizing radiation exposure to the fetus [10]. Unless concerning “red flag” features are present, treatment for sciatica generally begins with conservative care [11, 12]. Lumbar imaging is often recommended if patients fail to improve [13], however dedicated hip or pelvic imaging may ultimately be needed for atypical cases of sciatica originating outside of the lumbar spine [14–16].

Osteochondroma is a potential cause of sciatica, however, to our knowledge only three cases of sciatica resulting from ischial ramus osteochondroma have been reported [17–19]. Although osteochondroma is one of the most common benign bone lesions, these typically grow on long bones, with only 0.2% of osteochondromas growing from the ischium [20]. They are generally considered not to be true neoplasms, instead representing fragmentation of the epiphyseal growth plate that passes through the periosteal bone and continues to grow [21]. The majority of osteochondromas occur in the absence of hereditary multiple exostosis, are asymptomatic, and cease growing after skeletal maturity [21, 22]. Occasionally, osteochondromas cause symptoms through compression of neurovascular structures, formation of bursa, or fracture [21, 22]. Rarely, osteochondromas also may undergo malignant transformation, typically to become chondrosarcomas [22].

This case reports an ischial osteochondroma as an unusual cause of sciatic pain developing in the postpartum period. This case highlights diagnostic reasoning used to identify this rare cause of sciatica, with a chiropractor playing the key role in coordinating care.

### Case presentation

#### Patient information

**Fig. 1:**
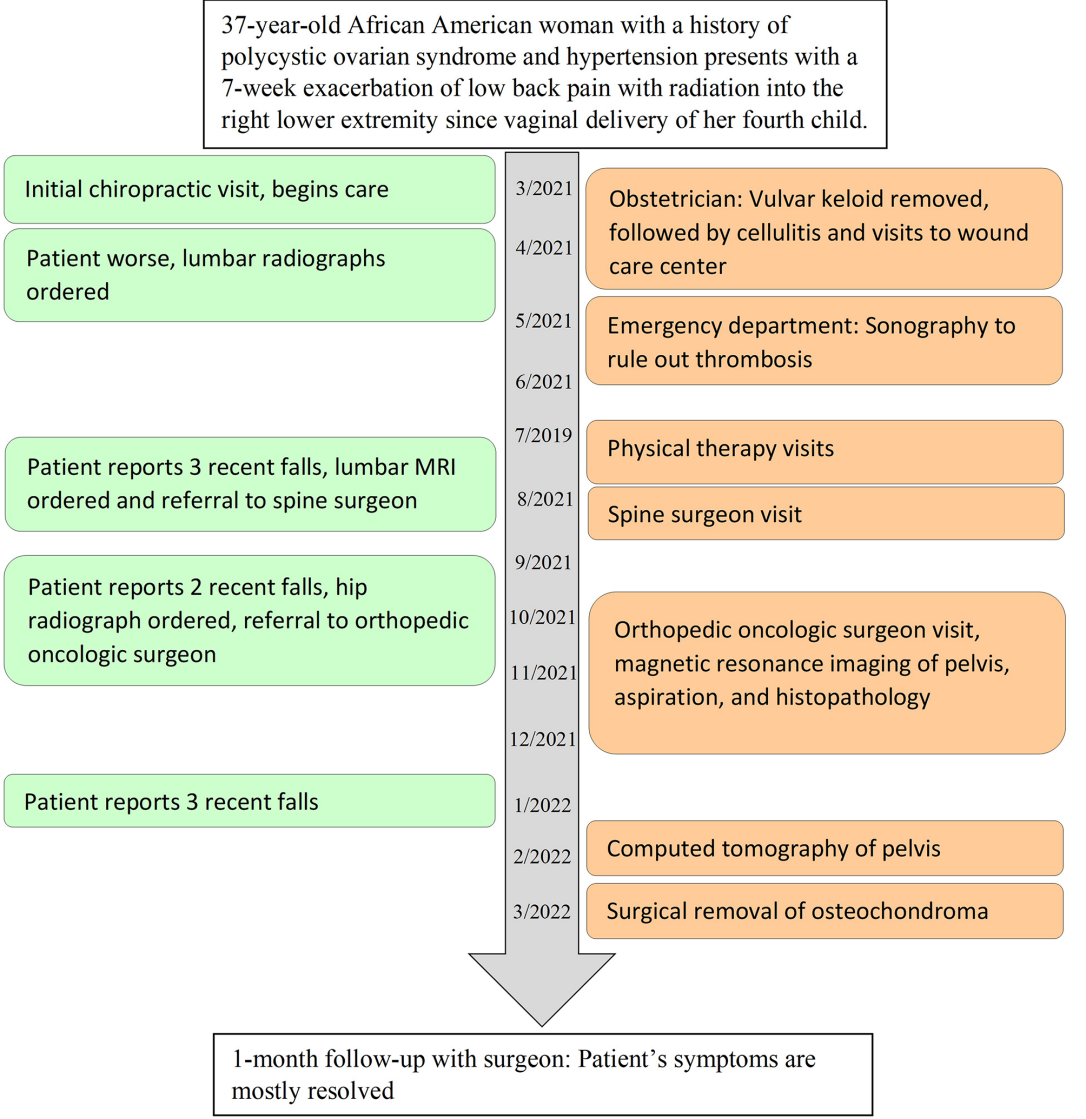
Timeline of events. Primary care physician (PCP), magnetic resonance imaging (MRI), computed tomography (CT), obstetrician (OB). The green boxes on the left detail major instances of chiropractic management, whereas orange boxes on the right detail care related to other providers

A 37-year-old African American woman, gravida four, para four, of 1.6 m and 81 kg (body mass index 30.6), presented to a chiropractor at a hospital-based outpatient clinic seven weeks postpartum (vaginal delivery at 36.6 weeks gestation). She was referred by her obstetrician for evaluation and management of low back and right gluteal pain with radiation into the posterolateral thigh, calf, and heel, with tingling in the foot, which she rated 5/10 in severity on the numeric pain rating scale (Fig. 1). The patient’s most recent pregnancy was achieved via in vitro fertilization, but complicated by preeclampsia and oligohydramnios. In addition, the patient underwent cervical cerclage at 16 weeks’ gestation due to having a short cervix and previous premature delivery. She noted having severe low back, hip, gluteal, and sciatic pain during her 24-hour duration of labor. Although she received epidural analgesia, it did not alleviate her low back, hip, gluteal, and sciatic pain. She had episodes of similar pain in previous years which were mild, occurring occasionally while driving. Pain was severe with sitting, driving, and when standing from a seated position, while ice provided some relief. The patient noted she had less than four hours of sleep per night due to pain.

The patient was a non-smoker, with occasional alcohol intake, who worked as a medical assistant. Her medical history was significant for polycystic ovary syndrome, keloid of the vulva, anxiety, for which she was prescribed 50 milligrams (mg) sertraline daily, and hypertension, for which she took 10 mg amlodipine daily. She had an intrauterine contraceptive device inserted one week prior to presentation. Ten years prior, she underwent laparoscopic surgery to remove left-sided ovarian cysts after suffering a cyst rupture, and also underwent bilateral tubal ligation. Fifteen years prior she had bilateral breast reduction surgery. Her family history was significant for ovarian cysts in her mother and diabetes mellitus, essential hypertension, malignant neoplasm of the colon, and prostate cancer in her father. There was no family history of hereditary multiple exostosis. Aside from the complicated recent labor, the patient denied having trauma to the low back or hip, bowel or bladder disturbances, fever, chills, and unintentional weight loss. The patient’s Oswestry Low Back Pain Disability Questionnaire score was 35% (moderate disability).

#### Clinical findings

On physical examination, the patient did not demonstrate any trunk list. However, she was noted to have a shortened stride length affecting the right lower extremity, limited by pain and tension during swing phase, and had slow guarded movements when transitioning from sitting to standing. Active lumbar range of motion was normal and did not exacerbate the patient’s pain, except for right rotation, which worsened her right low back and gluteal pain. Palpation revealed muscle hypertonicity and tenderness of the right gluteus medius and maximus, piriformis, and tensor fascia lata. Right hip extension and flexion were 4/5 strength (Medical Research Council Scale) with pain. There were no other deficits noted with sensory (i.e., light touch), motor, and reflex testing, and Babinski responses were downgoing.

Provocative special tests were performed bilaterally and only yielded abnormal findings on the right side. A right seated straight leg raise reproduced her lower extremity pain. Combined flexion, abduction, and external rotation of the right hip (i.e., FABER test) provoked right gluteal pain. A right lumbar quadrant test (Kemp’s) reproduced the patient’s right low back and gluteal pain. A right femoral nerve stretch test provoked local right hip pain.

The chiropractor’s initial differential diagnosis included lumbosacral radiculopathy caused by lumbar disc herniation, gluteal or piriformis tendinopathy affecting the sciatic nerve, and sciatic nerve neuropraxia related to lithotomy positioning or vaginal delivery. The patient was treated using high-velocity, low-amplitude thoracic spinal manipulation, lumbar flexion-distraction, and soft tissue manipulation, and was given home exercises to stretch the piriformis muscle.

**Fig. 2:**
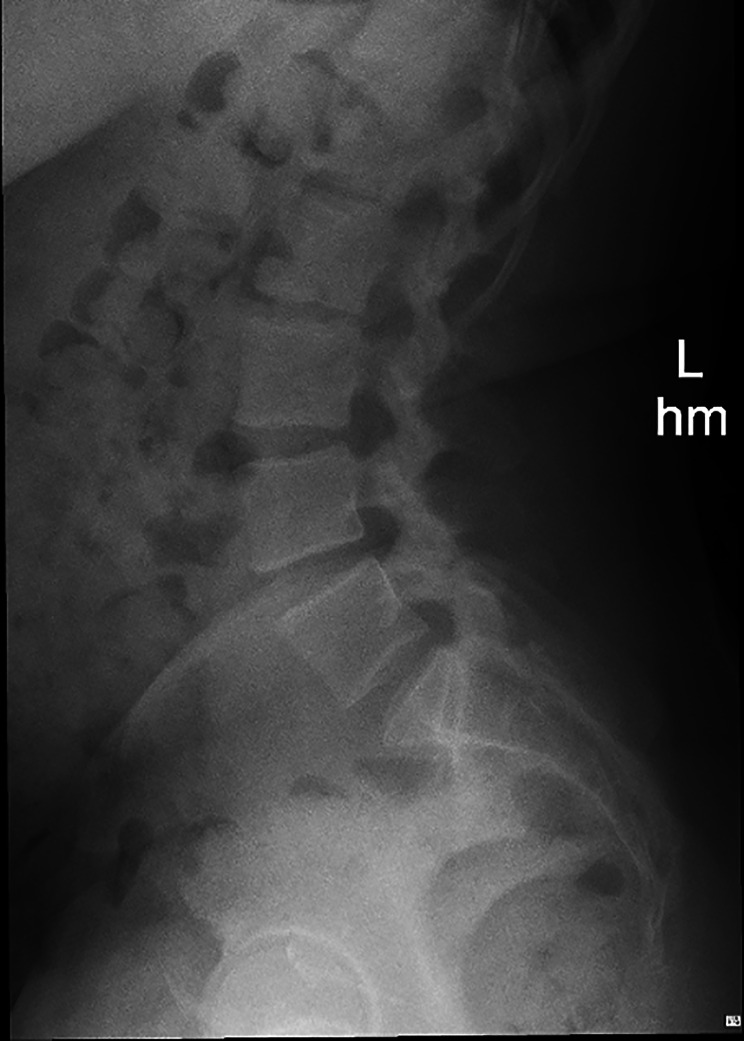
Lateral lumbar radiograph. This image and the anteroposterior view (not shown) showed a normal lumbar spine and were collimated above the level of the ischial tuberosity

One week after initial presentation, the patient underwent surgical removal of her vulvar keloid. She subsequently developed cellulitis and was prescribed 500 mg cephalexin daily. The patient returned to the chiropractor three weeks after initial presentation and reported worsening of lower extremity symptoms. She had begun using a walker occasionally due to her radiating pain and weakness. The chiropractor ordered lumbar radiographs, which were read as normal by a medical radiologist (Fig. 2), and referred the patient to a physical medicine and rehabilitation specialist. However, it was not feasible for the patient to visit this provider, as she was frequently attending a wound care center for her cellulitis, as referred by her obstetrician, as well as working and taking care of her four children.

Seven weeks after initial presentation, the patient returned for her third chiropractic visit. Manual therapy treatments including flexion-distraction were continued, and the patient noted short-term improvements in her low back pain but not her gluteal or sciatic symptoms. Nine weeks after presentation, the patient developed an exacerbation of right popliteal fossa pain and presented to urgent care and was referred to the emergency department. The emergency physician ordered venous Doppler sonography which ruled out deep vein thrombosis. The provider provisionally diagnosed a Baker’s cyst and referred the patient to physical therapy.

Sixteen weeks after presentation, the physical therapist identified hip weakness and painful, limited range of motion, Trendelenburg gait, and concurred with the possibility of Baker’s cyst, also suggesting potential lumbosacral radiculopathy. Treatment over three physical therapy sessions involved gluteal strengthening exercises such as gluteal bridges and clam shells. The patient reported that these exercises aggravated her symptoms and discontinued physical therapy. Upon returning to the chiropractor at 18 weeks after presentation, the patient reported three recent falls occurring when her right leg “gave out,” and returned to using a walker as needed. The chiropractor considered this a red flag of potentially serious pathology and accordingly referred the patient to a spine surgeon and ordered lumbar spine magnetic resonance imaging (MRI).

**Fig. 3:**
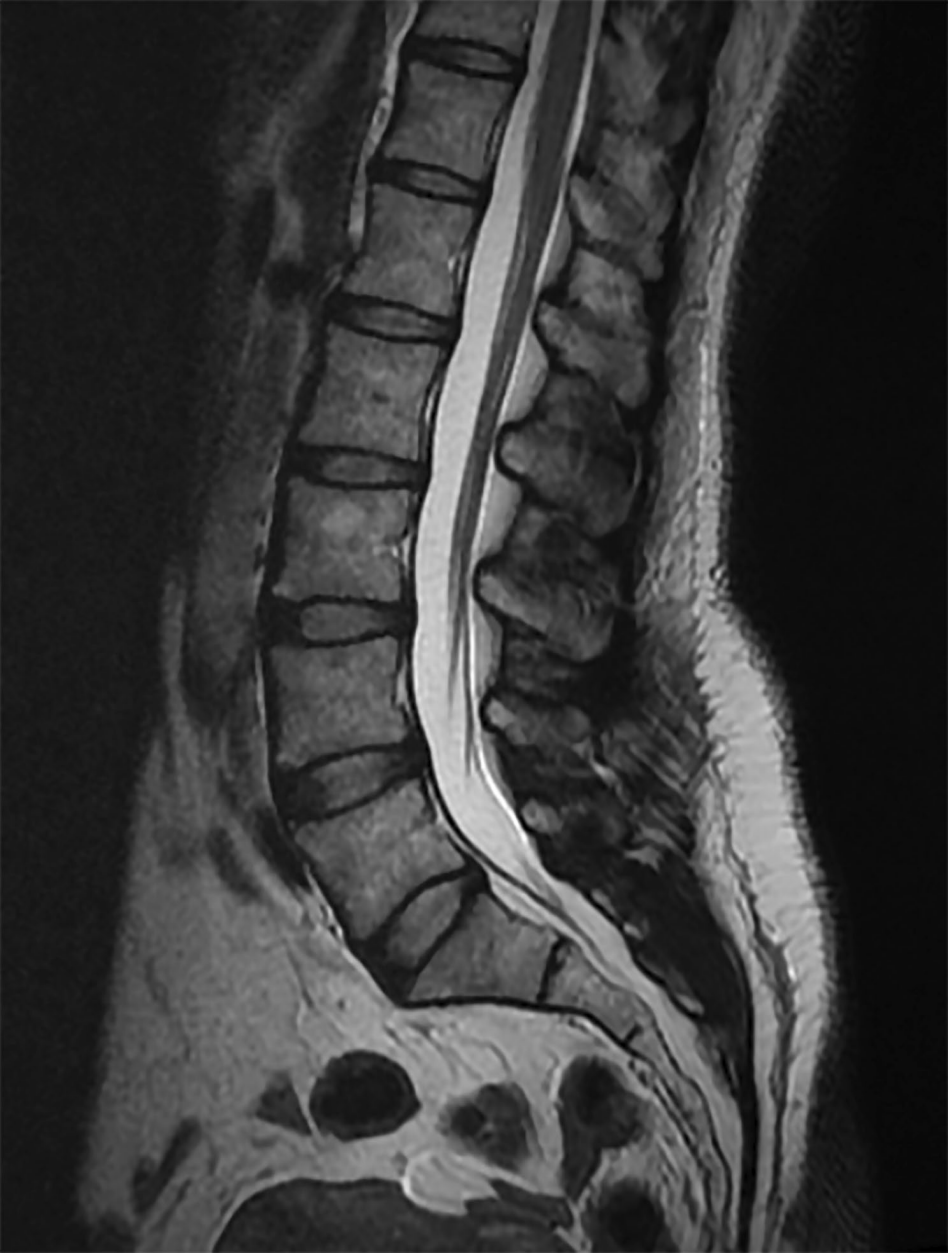
Lumbar spine MRI, T2 weighted non-fat saturated sequence, sagittal plane, representative image demonstrating normal lumbar spine

Twenty-one weeks after presentation, the patient presented to the spine surgeon noting gradual worsening of symptoms. The surgeon reproduced the patient’s symptoms with a right straight leg raise, and noted 4/5 strength of right knee extension, ankle dorsiflexion, ankle plantarflexion and extensor hallucis longus, and concurred with the plan to obtain lumbar MRI. The surgeon deemed the patient to not be a candidate for spine surgery, thus the chiropractor helped determine the next steps in management. Lumbar MRI was initially denied by the patient’s insurance company and was approved after an appeal, including a telephone peer-to-peer conversation with the chiropractor. The patient underwent lumbar MRI at 26 weeks, which was read as normal by a medical radiologist (Fig. 3).

**Fig. 4:**
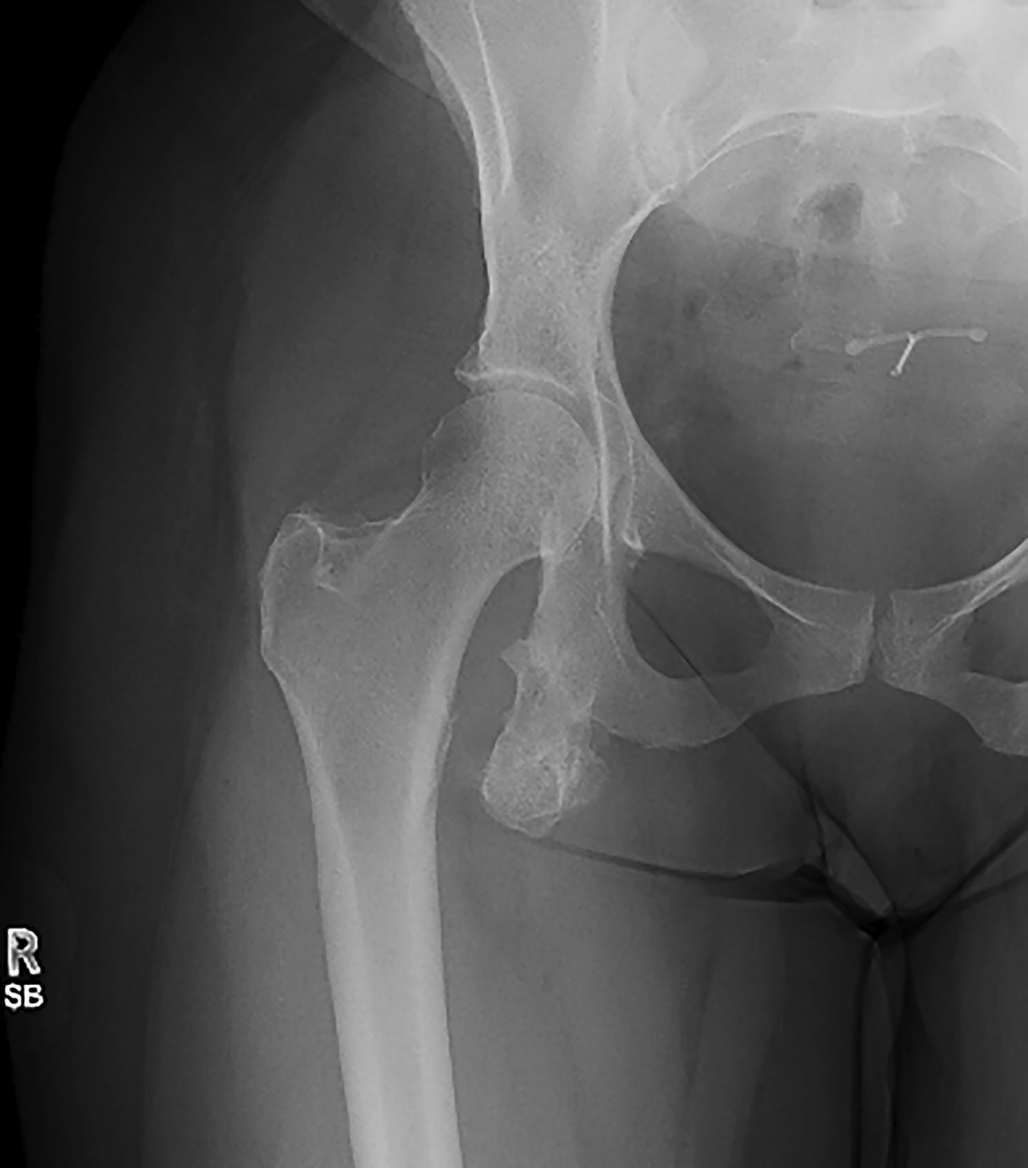
Anteroposterior right hip radiograph, demonstrating a large pedunculated bony excrescence arising from the right ischial ramus. The mass is seen to be continuous with the medullary canal and cortex of the ischium, a pathognomonic feature of osteochondroma. An intrauterine device is also visible within the pelvis

Twenty-nine weeks after presentation, upon returning to the chiropractor, the patient reported two additional falls. Considering the patient’s falls, normal lumbar MRI, and examination findings which failed to improve upon re-examination (i.e., gait abnormality, neural tension signs, and hip weakness) the chiropractor discarded the working diagnosis of radiculopathy. Instead, hip, pelvic, and sciatic nerve pathology were considered given the location of symptoms and timing of onset during labor. The chiropractor ordered hip radiographs with intentions of subsequently either ordering hip MRI or referring back to the obstetrician. At 31 weeks, hip radiographs read by a medical radiologist revealed a pertinent finding (Fig. 4) of “a large heterotopic ossification along the inferior aspect of [the] right ischial tuberosity.” The chiropractor provided a brief clinical summary to a small group of chiropractic colleagues within the department and asked for their help in reviewing the patient’s imaging and radiology report and determining next steps in care. After some research, the chiropractic team considered the imaging findings to potentially represent an osteochondroma and likely explain the patient’s sciatic symptoms. Accordingly, the chiropractor referred the patient to an orthopedic oncologist.

**Fig. 5:**
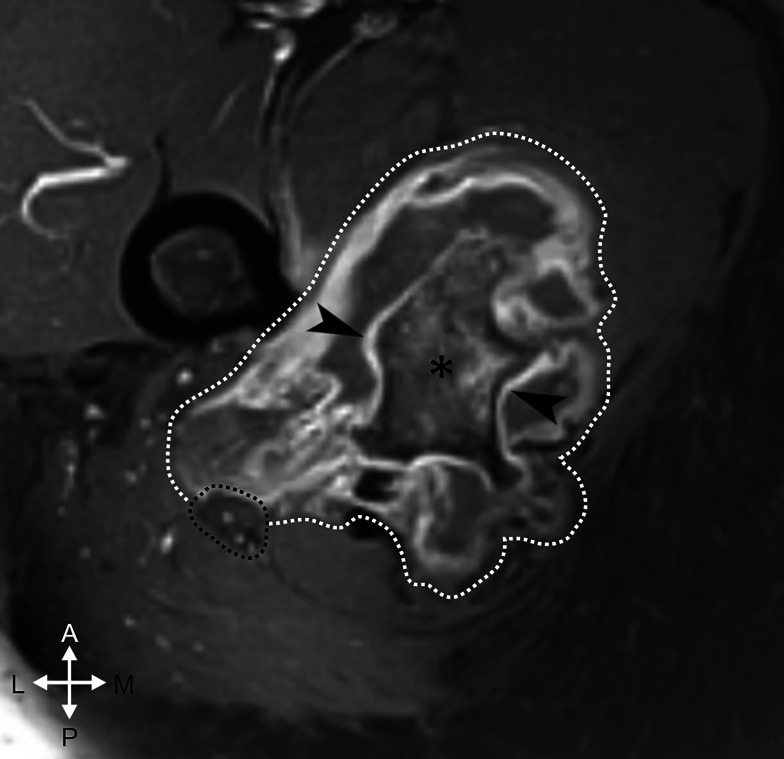
Pelvis MRI, T1 weighted fat saturated sequence with gadolinium-based contrast, axial plane, of the right hip just inferior to the level of the lesser trochanter. The osteochondroma (*) displays a thin post contrast T1 hyperintense rim representing the fibrovascular tissue overlying the thin cartilaginous cap (arrowheads). The overlying adventitial bursa wall is post contrast T1 hyperintense (white dotted line) while the bursal contents are of an intermediate T1 signal intensity, less than skeletal muscle, likely related to its serosanguineous contents. The sciatic nerve (black dotted line) is displaced posteriorly and laterally from its normal course. Anterior (A) and posterior (P), and medial (M) and lateral (L) orientation is noted. Corresponding T2 fat saturated and T1 non fat saturated pre contrast axial slices are attached within the Supplemental File, (Supplemental Fig. 1, Supplemental Fig. 2)

The following week, the patient visited the orthopedic oncologist who noted the presence of a palpable firm fixed fullness in the inferior right buttock. This provider ordered pelvic MRI without and with gadolinium-based contrast, which, at 32 weeks identified a large pedunculated osseous excrescence measuring 5.2 × 1.3 centimeters (cm; craniocaudal x mediolateral) arising from the right ischial tuberosity with corticomedullary continuity, which was favored to represent an osteochondroma (Fig. 5). The right sciatic nerve was enlarged, with a cross sectional area of 54 millimeters^2^ (mm) compared to the left sciatic nerve which was 25 mm^2^. Also noted was an overlying lobulated fluid collection, favored to represent a large reactive adventitial bursitis. The radiologist considered the possibility of an atypical morphology of the cartilaginous cap, and recommended correlation with histopathological sampling.

After discussion of treatment options with the patient, there was no rush to excise the lesion considering it had likely been present for several years without major symptoms until recently. The patient instead elected for ultrasound-guided aspiration and injection as a minimally invasive treatment in an attempt to avoid surgical intervention. The ultrasound and aspiration had the additional purpose of determining the possibility of malignant degeneration, by verifying the presence of the adventitial bursa rather than an atypical thick cartilage cap, and to assess for any cellular atypia in the aspirate.

Thirty-five weeks following the initial presentation, sonography demonstrated a cortical excrescence abutting an anechoic fluid collection. Given that there was no soft tissue component present, aspiration of the collection with subsequent steroid and anesthetic injection was performed as opposed to biopsy. 1% lidocaine was injected subcutaneously and then into the deeper tissues surrounding the fluid collection, and 35 cubic cm of serosanguineous fluid was aspirated. Ultrasound demonstrated resolution of the fluid collection. Then, a combination of two anesthetic agents, bupivacaine and lidocaine, and a glucocorticoid, triamcinolone (Kenalog), was injected into the site where the fluid collection was located. A smear of the aspirated material with a gram stain identified fibrous tissue and 2 + granulocytes, with no pathogenic organisms. A fluid culture did not detect growth aerobically or anaerobically after three days.

Thirty-seven weeks after presentation, the patient returned to the orthopedic oncologist, reporting improvement since the fluid aspiration and injection. She preferred to undergo careful monitoring with follow-up computed tomography instead of surgery. However, her low back pain was further exacerbated by three additional falls, prompting her to return to the chiropractor at 45 weeks. Her Oswestry Low Back Pain Disability Questionnaire score had worsened to 40%. Additional hip radiographs had been performed at a local emergency department which were negative for fracture of the osteochondroma. Chiropractic treatments at this point afforded the patient some relief from the acute low back pain caused by the falls, which appeared to affect the sacroiliac joints bilaterally based on location and provocative testing.

**Fig. 6:**
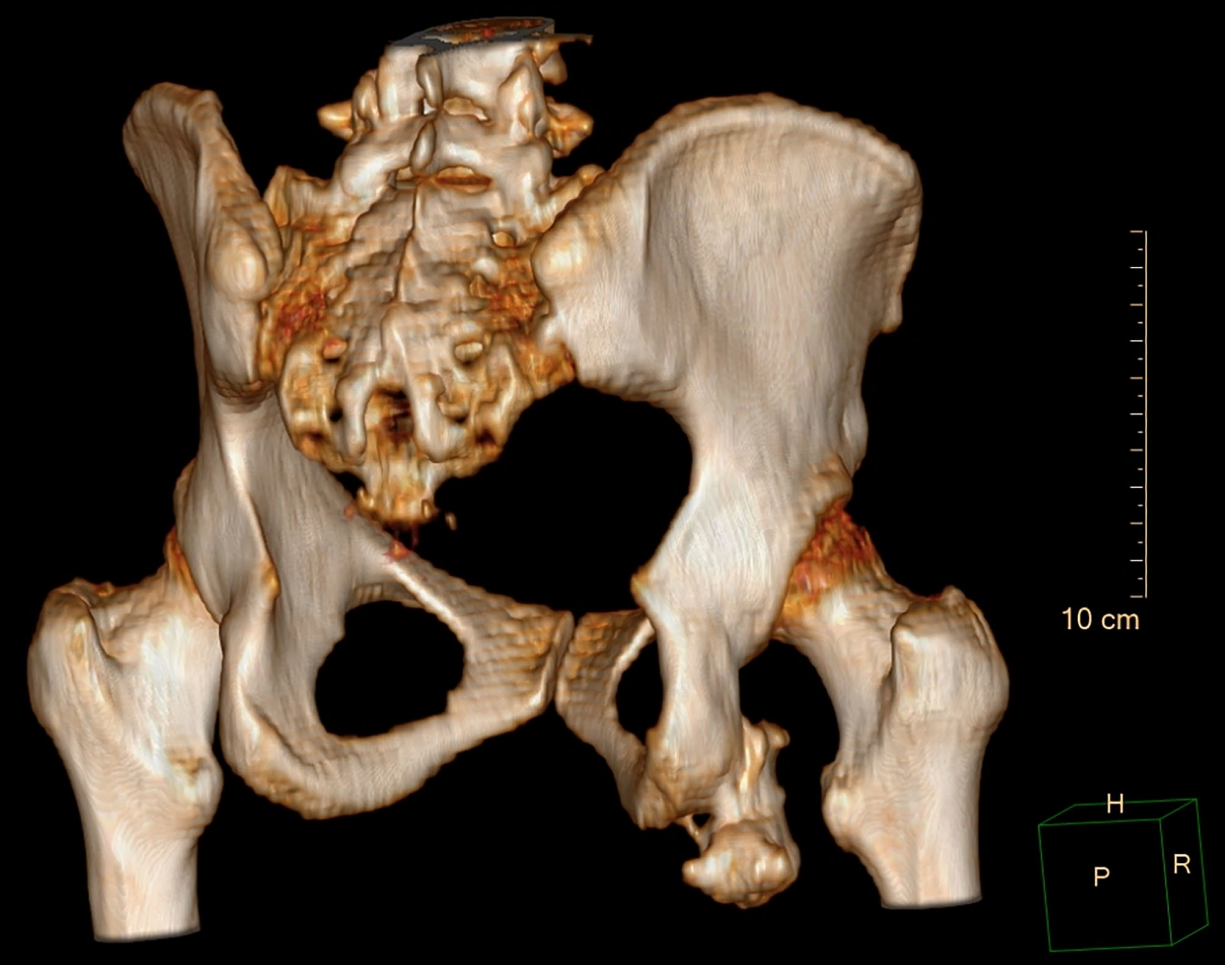
Three-dimensional reconstruction of the pelvis, posterolateral view. The osteochondroma is visible at the lower right side of the image, attached to the ischial ramus. The distance between the osteochondroma and lesser trochanter is 11 millimeters. Orientation of head (H) right (R) and posterior (P) and dimensions in centimeters (cm) are noted

Forty-seven weeks after presentation, computed tomography demonstrated a large pedunculated osteochondroma arising from the right ischial tuberosity (Fig. 6), again re-demonstrating an adventitial bursitis extending into right ischiofemoral space. At 48 weeks, the patient made an informed decision to pursue surgery, which was performed at 52 weeks.

**Fig. 7:**
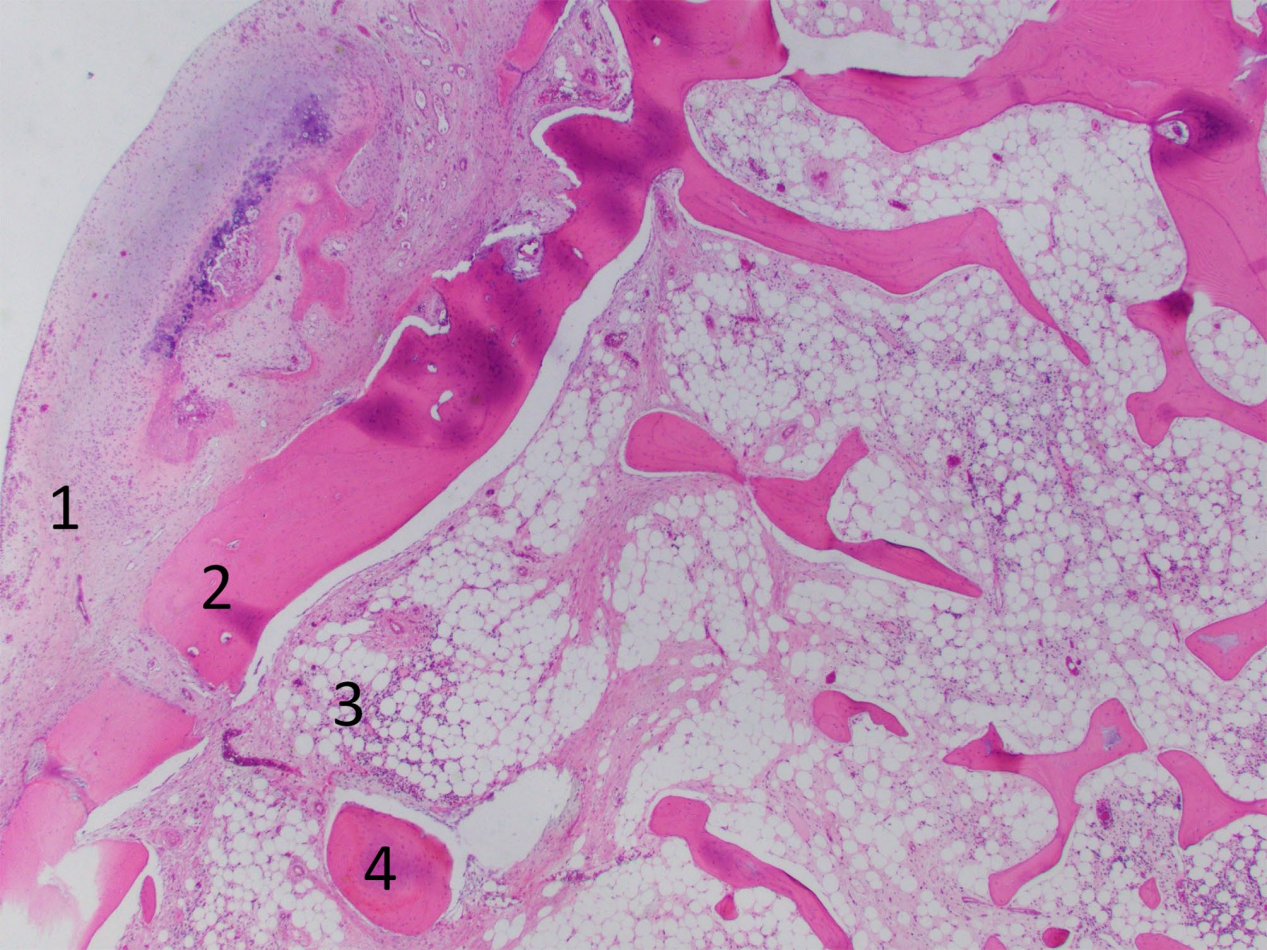
Hematoxylin and eosin section shows a thin, 2-millimeter cartilage cap (1) with an underlying cortical rim (2), marrow space (3), and several bone trabeculae (4). 20 x magnification

The surgeon utilized a posterior hip (Kocher-Langenbeck) approach to access the osteochondroma, which involved using the inferior two-thirds of an incision angled from the greater trochanter towards the posterior superior iliac spine. The sciatic nerve was found to be displaced over the posterior aspect of the osteochondroma and at this point was erythematous and edematous, and was protected and retracted medially. Two osteotomes were used to make an osteotomy at the base of the osteochondroma, leaving a small amount of osteochondroma bone to decrease the likelihood of a stress riser fracture. The osteochondroma was then released from its soft tissue attachments. The remaining osteochondroma was removed with a rongeur and then smoothed with a rasp. Once the lesion was removed the sciatic nerve moved freely. An intraoperative x-ray showed that the majority of the lesion was removed. Histologic examination of the lesion revealed a lobulated segment of bone with a white cartilaginous surface measuring 0.2 cm (Fig. 7).

**Fig. 8:**
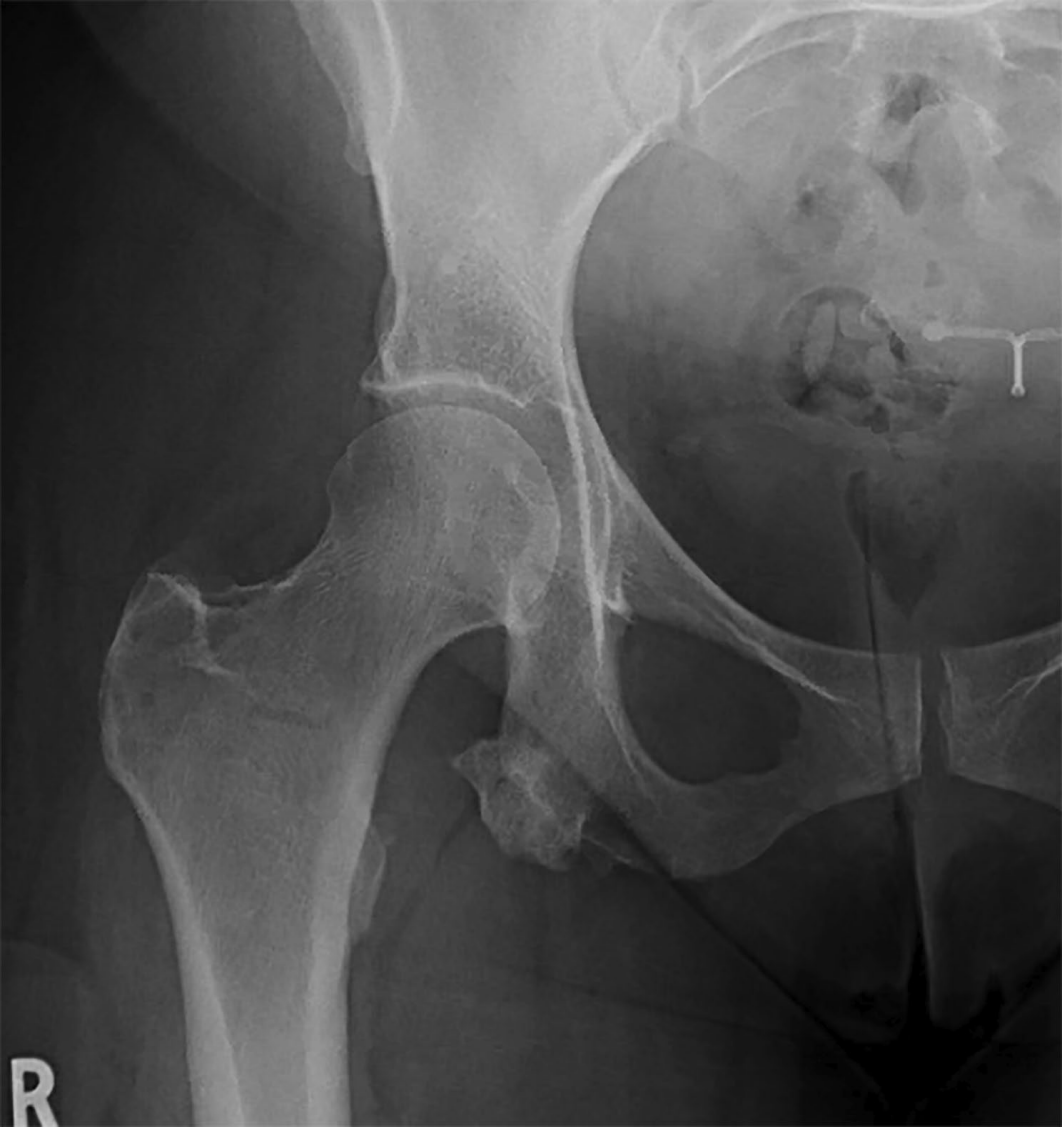
One-month postoperative anteroposterior hip radiograph revealing a significant reduction in size of the ischial tuberosity osteochondroma. Residual osteochondroma was intentionally left to reduce the chances of a post-operative stress riser fracture

At the post-operative visit with the surgeon, eight days post-surgery, the patient reported a resolution of her preoperative low back pain and radiating sciatic pain, however there was local pain and soreness in the gluteal and hip area. The patient also noted improvements in gait and no longer had falls or required the use of a walker. At her one-month post-surgery visit with the surgeon she underwent a routine follow-up radiograph (Fig. 8), and reported her only symptoms were mild intermittent posterior right knee pain. Six weeks after surgery the patient returned to the chiropractor for a follow-up and noted mild localized low back pain without radiation, 3/10 severity on a numeric pain rating scale. On examination there were no strength deficits in the lower extremity. The patient provided written informed consent for the publication of this case report and any accompanying images.

## Discussion and conclusions

Upon initial presentation, the patient displayed common features of radicular sciatica. However, the chiropractor discarded this diagnosis after lumbar imaging was normal in the presence of worsening symptoms, and subsequently evaluated the hip. Obstetric palsy became less likely during the course of care, considering this typically resolves over a median of two months [4]. While a Baker’s cyst was suggested, this would not explain the patient’s more proximal symptoms, weakness, and falls. Referral to an orthopedic oncologist helped uncover the diagnosis in this case, an ischial ramus osteochondroma with adventitial bursitis causing sciatic neuropathy. Accordingly, any initial transient relief with chiropractic treatments may have been related to alleviation of secondary musculoskeletal sources of low back pain.

After the sciatic nerve exits the pelvis it normally descends through the ischiofemoral space, the region between the ischial tuberosity and femur [23]. This space is normally two cm in the transverse plane, yet when narrowed or impinged upon, may compress muscles, tendons, and the traversing sciatic nerve. Common causes of ischiofemoral impingement include variations in anatomy and muscle injuries [23]. Tumors and tumor-like conditions are occasionally reported, including osteochondromas of the lesser trochanter [23–25], and ischial tuberosity as described in the current case.

In the current case, the osteochondroma narrowed the ischiofemoral space to roughly half of its typical measurement, and, along with bursitis, displaced and tethered the sciatic nerve. This mechanism likely explained the neural tension symptoms with leg raising. Hip flexion also narrows the ischiofemoral space [26], which could explain the patient’s exacerbation in hip flexion positions during examination, as deep hip muscle, tendons, and adventitial bursa were likely compressed. Further, pain with sitting and a shortened stride are consistent with ischiofemoral impingement [23].

The timing of symptom onset during labor was likely linked to mechanical factors, rather than growth of the osteochondroma which would be unlikely as the patient had already reached skeletal maturity. Prolonged hip flexion during the lithotomy position may further stretched the sciatic nerve and narrowed the ischiofemoral space, which were already compromised at baseline from the osteochondroma. We suspect this labor position was responsible for the abrupt exacerbation of sciatic symptoms.

The current case is also interesting in that the ischial ramus osteochondroma was associated with a large adventitial or reactive bursitis. While this type of bursitis has been reported with osteochondromas in other locations [27], it has not been described among previous cases of ischial osteochondroma, to our knowledge [17–19, 28]. It is thought that the osteochondroma irritates neighboring structures via friction, leading to an inflammatory process which can be complicated by hemorrhage [27]. These elements were likely present in the current case, as evidenced by adventitial bursa formation which contained serosanguinous fluid with granulocytes at fluid analysis.

This case illustrates the role of a chiropractor in an integrative setting with regards to coordinating care. This involved ordering radiographs and MRI and placing formal referrals via the shared electronic health records system to physical medicine, spine surgery, and orthopedic oncology, each with customized clinical summaries explaining the rationale for the requested visit and current differential diagnosis. The chiropractor was in close contact with the patient via a secure messaging system (i.e., patient portal) which facilitated obtaining appointments and tracking her progress before and after surgery. A 2019 survey reported that only 5% of chiropractors in the United States have a hospital affiliation [29]. Chiropractors not only perform manual therapies, but are trained to take a patient history, conduct an examination, make referrals, and order imaging as needed [30, 31]. These steps were all relevant to the positive outcome in the current case. However, as a single case report, the findings in this case may not be broadly generalizable.

This case describes an ischial ramus osteochondroma as a rare cause of sciatica developing during labor. Misleading symptoms and other challenges led to a significant diagnostic delay in this case, and an accurate diagnosis was only made after placing greater attention to the following key features: (1) Symptom onset during labor, (2) worsening despite conservative care, and (3) normal lumbar spine imaging. Clinicians should consider atypical sources of sciatica outside of the lumbar spine when symptoms arise during labor, are refractory to conservative care, and lumbar imaging is normal.

## Electronic supplementary material

Below is the link to the electronic supplementary material.

Supplementary Material 1

## Data Availability

All data generated or analysed during this study are included in this published article and its supplementary information files.

## Declarations

### Abbreviations

Anterior: A; Centimeters: cm; Computed tomography: CT; Head: H; Lateral: L; Magnetic resonance imaging: MRI; Medial: M; Millimeters: mm; Mg: milligrams; Obstetrician: OB; Posterior: P; Primary care physician: PCP; Right: R
