# Supplementary material for: Ischial osteochondroma as an unusual source of pregnancy-related sciatic pain: a case report
Source: Chiropr Man Therap. 2022 Oct 17;30:45. doi: 10.1186/s12998-022-00451-3 (PMC9575271; doi:10.1186/s12998-022-00451-3)
Supplement: Supplementary file 1 — Supplementary Material 1 [file 12998_2022_451_MOESM1_ESM.docx]

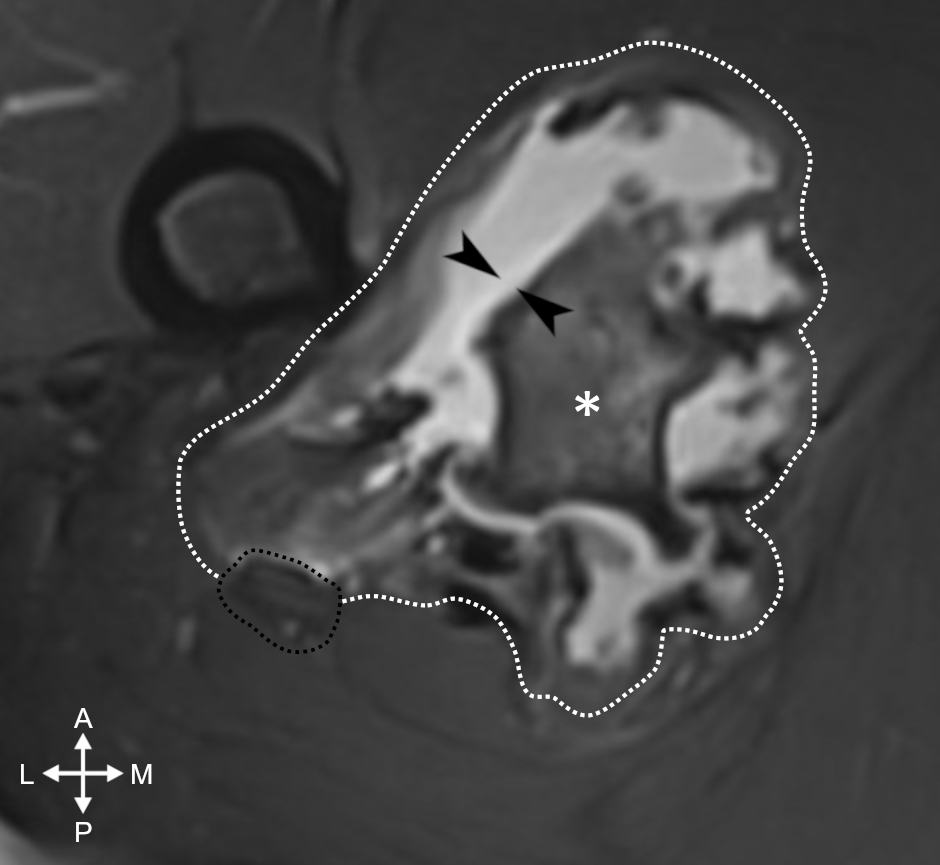


Supplemental Figure 1: Pelvis MRI, T2 weighted fat saturated sequence, axial plane, of the right hip just inferior to the level of the lesser trochanter. The osseous portion of the osteochondroma (*) is seen centrally showing internal characteristics of medullary bone, while there is only subtle appreciation of the high T2 signal intensity cartilage cap (arrowheads) at the bursal fluid interface. The overlying adventitial bursa (white dotted line) is hyperintense. The sciatic nerve is displaced posteriorly and laterally from its normal course (black dotted line). Anterior (A) and posterior (P), and medial (M) and lateral (L) orientation is noted.


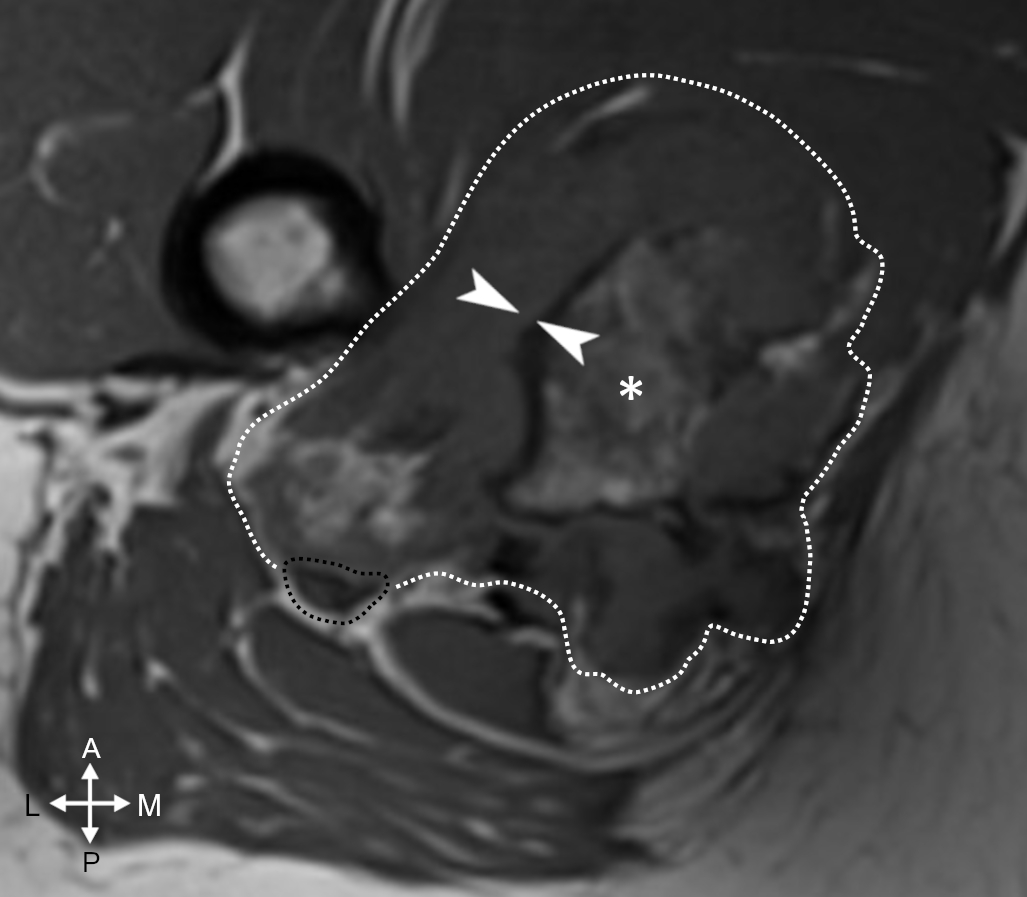


Supplemental Figure 2: Pelvis MRI, T1 weighted non fat saturated sequence, axial plane, of the right hip just inferior to the level of the lesser trochanter. The osseous portion of the osteochondroma (*) is seen centrally, showing internal characteristics of medullary bone and the overlying T1 hypointense cartilage cap is only subtly appreciated (arrowheads). The overlying adventitial bursa (white dotted line) is isointense to slightly hyperintense to skeletal muscle. The sciatic nerve (black dotted line) is displaced posteriorly and laterally from its normal course (white dotted line). Anterior (A) and posterior (P), and medial (M) and lateral (L) orientation is noted.
